# Complete Genomic Analysis of VRE From a Cattle Feedlot: Focus on 2 Antibiotic Resistance

**DOI:** 10.3389/fmicb.2020.571958

**Published:** 2020-10-15

**Authors:** Frank Eric Tatsing Foka, Charlotte Mienie, Cornelius Carlos Bezuidenhout, Collins Njie Ateba

**Affiliations:** ^1^Food Security and Safety Niche Area, Faculty of Natural and Agricultural Sciences, North-West University, Mmabatho, South Africa; ^2^Unit for Environmental Sciences and Management, North-West University, Potchefstroom, South Africa

**Keywords:** vancomycin-resistant enterococci, *E. durans* strain NWUTAL1, *E. gallinarum* strain S52016, whole-genome sequencing, food safety

## Abstract

Practices in intensive animal farming such as the extensive use of antimicrobials have significant impacts on the genetic make-up of bacterial communities, especially on that of human/animal commensals. In this report, whole genome sequencing of two vancomycin-resistant enterococci (VRE) isolates from a cattle feedlot in the North West Province, South Africa, was used to highlight the threats that extensive antimicrobial usage in intensive animal rearing represents for environmental microbiomes and the food chain. The genomic DNA of the studied strains was extracted using a DNA extraction kit. Whole-genome sequencing was performed through next-generation sequencing. The genomes of *Enterococcus durans* strain NWUTAL1 and *Enterococcus gallinarum* strain S52016 consisted of 3,279,618 and 2,374,946 bp, respectively with G + C contents of 40.76 and 43.13%, respectively. Antibiotic resistance genes (ARG), plasmids and virulence factors (involved in biofilm formation, colonization and copper/silver efflux system), were detected in the genomes of both strains. The presence of these genetic determinants in the studied strains is a cause for concern as they may disseminate and find their way into the food chain via horizontal gene transfer amongst bacteria of the different ecological niches. Issues of this nature cannot be undermined and are relevant as far as food safety is concerned.

## Introduction

The discovery of antibiotics was a significant hallmark in the evolution of mankind as they became important life-saving compounds both for animals and humans (Gonzalez-Zorn and Escudero, 2012). In fact, antimicrobials have impacted significantly on society and the health of humans and animals mainly because life expectancy could be ameliorated as common infections have become curable, thus promoting rapid growth of the population (Gonzalez-Zorn and Escudero, 2012). Unfortunately, as the therapeutic effects of antibiotics were discovered, their growth-promoting attributes became apparent, resulting in the extensive use of these agents as growth promoters in intensive animal rearing (Acar et al., 2012; Economou and Gousia, 2015). According to Marshall and Levy (2011), subtherapeutic doses of certain antibiotics that are used as growth promoters improve feed conversion, animal growth and diminish mortality and morbidity rates that arise from clinical and subclinical diseases. However, the mechanism through which this is achieved is unclear (Marshall and Levy, 2011). Consequently, multidrug resistant isolates have emerged not only because of the abusive use of antibiotics/antimicrobials in communities and clinics, but mostly because of widespread use of antimicrobials in industrial animal farming (Boxall et al., 2002; Acar et al., 2012). However, there are studies that highlight that resistant bacteria and resistance mechanisms were present long before antibiotics were produced or used in clinical practise (Boxall et al., 2002; Acar et al., 2012).

A significant consequence of the widespread use of antibiotics in industrial animal farming is the presence of genetic resistance determinants in the environment and its ecological niches (Ding et al., 2014). This also results from the fact that antibiotics are not totally degraded into inactive compounds in the body of treated animals and excreted with feces in manure where they regain their initial molecular structure after some time (Forsberg et al., 2014). The manure becomes a hotspot for resistance determinants, which when mixed with soil, genetic material is transferred to other bacteria of the soil (Forsberg et al., 2014; Thanner et al., 2016). Moreover, as a result of agricultural lands runoffs, water bodies become contaminated with resistant strains that exchange genetic material with other commensals present in the water bodies which may eventually find their way into the food chain (Economou and Gousia, 2015).

Enterococci are commensals of the gastrointestinal tract of warm-blooded animals. Enterococci have the ability to cause illnesses both in animals and immunocompromized individuals. In fact, they can cause endocarditis, septicemia, urinary tract infections, burn wound and deep tissue infections in humans meanwhile they are responsible for intramammary infections and clinical mastitis in cattle (Myllys and Rautala, 1995; Bager et al., 1999; Aarestrup et al., 2000). Vancomycin-resistant enterococci (VRE) emerged four decades ago due to the misuse of avoparcin (a glycopeptide analog of vancomycin) as a growth promoter in intensive animal rearing and the abuse of vancomycin in clinics for the therapeutic management of community-acquired enterococcal infections (Myllys and Rautala, 1995; Bager et al., 1999). Since then, avoparcin has been banned in intensive animal farming (Bager et al., 1999). However, the constant detection of VRE worldwide (Arthur et al., 1996; Depardieu et al., 2004; Courvalin, 2006; Sundermann et al., 2019; Tatsing and Ateba, 2019) is indicative of the fact that factors other than avoparcin may be the source of the dissemination of VRE in the environment. Resistance to vancomycin can be either intrinsic or acquired. Eight types of vancomycin resistance gene clusters have been characterized so far (*vanA*, *vanB*, *vanC*, *vanD*, *vanE*, *vanG*, *vanL*, *vanM*, and *vanN*) (Depardieu et al., 2004).

Although there are several studies on the detection of antibiotic resistant strains such as VRE worldwide (Myllys and Rautala, 1995; Arthur et al., 1996; Depardieu et al., 2004; Courvalin, 2006; Tatsing and Ateba, 2019), there is a need to investigate the possible effects that practices such as the misuse of antimicrobials/antibiotics in industrial animal farming facilities, have on the genetic constitution of environmental bacteria and, consequently, on the different ecological niches of the environment and the food chain. A decade ago, whole genome sequencing (WGS) technologies were introduced in epidemiological studies, thus generating huge amounts of relevant data. WGS has been used since then to decode the genetic constitution of a considerable number of enterococcal species from various sources, thereby putting in the spotlight, genetic determinants involved in antibiotic resistance as well as those involved in pathogenesis processes which were previously less studied (Rangel et al., 2019; Sundermann et al., 2019). As WGS tools were gradually used in epidemiological investigations, *Enterococcus faecium* and *Enterococcus faecalis* have become the most studied enterococci, disregarding other supposedly harmless species such as *Enterococcus durans* and *Enterococcus gallinarum*, which have evolved into highly resistant strains with time (Rogers et al., 1992; Taucer-Kapteijin et al., 2016; Tatsing and Ateba, 2019). Since *E. durans* and *E. gallinarum* are mostly associated with environmental samples, less focus has been given to these species, due to which their whole genomic data are insufficient as compared to those of *E. faecalis* and *E. faecium* strains (Rogers et al., 1992; Jenney et al., 2000).

The aim of the study was to analyze the whole genomes of two vancomycin-resistant enterococcal strains, specifically *E. durans* NWUTAL1 and *E. gallinarum* S52016 isolated from a feedlot (cattle feces and soil, respectively) and further, demonstrate the impact of antimicrobial usage in animal farming on the genetic constitution of these strains (by evaluating their genomic diversity as well as their resistome) and the risk that such strains represent for food safety.

## Materials and Methods

### Bacterial Strains

Two vancomycin-resistant strains, *E. durans* NWUTAL1 was recovered from fecal samples obtained from cattle while *E. gallinarum* S52016 was recovered from samples obtained from feedlot soil in Rooigrond, North-West Province, South Africa (Tatsing and Ateba, 2019), and stored at −80°C in Luria-Bertani broth supplemented with 50% (v/v) glycerol. These isolates were resistant to tetracycline (TET–30 μg), ampicillin (AMP–10 μg), amoxicillin (AMX–10 μg), vancomycin (VAN–30 μg), penicillin (PEN–10 μg), linezolid (LIN–30 μg), and erythromycin (ERY–15 μg) thus the multi-drug phenotypes were TET^R^-AMP^R^-AMX^R^-VAN^R^-PEN^R^-LIN^R^-ERY^R^. They also harbored resistant determinants *vanA*, *vanB*, *vanC*, *tetK*, *tetL*, *msrA/B*, and *mefA* as well as the virulence genes *cylA*, *hyl*, *esp*, *gelE*, and *asa1*. The identities of the *E. durans* strain NWUTAL1 and *E. gallinarum* strain S52016 were confirmed in a previous study (Tatsing and Ateba, 2019; Tatsing, 2020) and their 16S rRNA gene sequences were deposited in GeneBank with accession numbers MK086097 and MK086099, respectively.

### Genomic DNA Extraction and Detection of Vancomycin-Resistant Enterococci (VREs)

Pure *E. durans* strain NWUTAL1 and *E. gallinarum* strain S52016 colonies were revived by sub-culturing on nutrient agar. Pure colonies were inoculated in 20 ml brain heart infusion broth (BHI, Merck, South Africa) and incubated overnight at 37°C. Bacteria cells were harvested through centrifugation. Genomic DNA was extracted with a DNA extraction kit (Zymo Research Genomic DNATM–Tissue MiniPrep Kit, ZR Corp. Irvine, United States) and quantified using a NanoDrop TM 1000 spectrophotometer (Thermo Fischer Scientific, United States).

### Sequencing and Library Preparation of Whole Genome

The draft genomes were obtained through WGS using an Illumina Miseq platform. 1 ng of the genomic DNA was tagmented with the Nextera XT DNA library prep kit according to the manufacturer’s protocol. The kit reagents fragment the DNA with simultaneous addition of adapter sequences. The libraries were amplified with a limited-cycle PCR program (12 cycles) to add the index 1 (i7) and index 2 (i5) adapters, containing sequences required for cluster generation of the Illumina flow cell. The library was purified using 0.6× Agencourt AMPure XP beads (Beckman Coulter). The quality and sizes of the resulting DNA fragments were evaluated on a 1.5% (w/v) agarose gel. The libraries were quantified with a fluorometric method (Qubit, Life Technologies) and normalized to 4 nM using a standard dilution method. The libraries were pooled, denatured with 0.1 N NaOH and diluted to the final loading concentration of 12 pmol. An identically treated PhiX Control v3 adapter-ligated library at low-concentration spike-in of 1% was added as an in-lane positive control for alignment calculations and quantification efficiency. Paired-end sequencing was done on an Illumina MiSeq platform using a MiSeq Reagent Kit V3 600 cycles.

### Sequence Quality Checking, Trimming and Assembly

Sequence data from Illumina platform were extracted and uploaded on Kbase. The quality of the raw sequences reads were assessed with FastQC (v0.11.5) (Wingett and Andrews, 2018). Low quality sequences and adapters were removed with Trimmomatic (v0.36) (Bolger et al., 2014). The sequences reads were *de novo* assembled using SPAdes (v3.13.0) (Bankevich et al., 2012).

### Genome Annotation and Comparative Analysis

The genomes of our strains of interest were annotated using Prokka (v1.12) (Seemann, 2014), RAST (v0.11) (Overbeek et al., 2014) and the NCBI prokaryotic genome annotation pipeline (Tatusova et al., 2015). Algorithms of the Pathosystems Resource Integration Center (PATRIC 3.5.41) (Wattam et al., 2017), ResFinder (v3.1.0) (Zankari et al., 2012) and PlasmidFinder (v2.0) (Carattoli et al., 2014) were used to assess the resistome, plasmids and virulence factors in the draft genomes. The Genome Annotation Service in PATRIC uses k-mer-based Antibiotic resistance genes (ARG) detection method, which utilizes PATRIC’s curated collection of representative ARG sequence variants and assigns to each ARG, functional annotation, broad mechanism of antibiotic resistance, drug class and, in some cases, specific antibiotic it confers resistance to. CGView server was used to generate a circular map of the genomes (Grant and Stothard, 2008). The phylogenetic relationships with other strains of the respective species of interest were also assessed with PATRIC (v3.5.41) (Wattam et al., 2017). Finally, the presence of clustered regularly interspaced short palindromic repeats (CRISPR) and bacteriophages in the draft genomes of interest were assessed with CRISPRFinder (Grissa et al., 2007) and PHASTER (Arndt et al., 2016).

Reference genomes from NCBI were used by PATRIC algorithms to generate a phylogenetic tree. The closest reference and representative genomes to our strains of interest were identified by Mash/MinHash (Ondov et al., 2016). PGfams were selected from these genomes to determine the phylogenetic placement of our genomes of interest. The protein sequences from these families were aligned with MUSCLE (Edgar, 2004), and the nucleotides for each of these sequences were mapped to the protein alignment. The joint set of amino acid and nucleotide alignments were concatenated into a data matrix, and RaxML (Stamatakis, 2014) was used to analyze this matrix with fast bootstrapping (Stamatakis et al., 2008) in order to generate the support values in the phylogenetic tree.

### Data Analysis

Statistica 13 (StatSoft, TIBCO software Inc., United States) was utilized to organize and interpret the data generated in this study.

## Results

### Genomic Assembly Features of *E. durans* NWUTAL1 and *E. gallinarum* S52016

VR *E. durans* Strain NWUTAL1 was recovered from fecal samples obtained from cattle while VR *E. gallinarum* strain S52016 was recovered from samples obtained from feedlot soil. Both strains possessed *vanC* resistance gene and their genome sequences were submitted to NCBI GenBank. Data derived from the assembly and the annotation of the genomes studied are summarized in Table 1. The genomes have 3,517 versus 2,351 protein coding sequences, respectively, 59 versus 30 transfer RNA sequences, respectively and 4 versus 5 ribosomal RNA sequences, respectively for strains NWUTAL1 and S52016.

**TABLE 1:** Assembly reports of *E. durans* NWUTAL1 and *E. gallinarum* S52016 genomes.

**Features**	***E. durans* NWUTAL1**	***E. gallinarum* S52016**
Genome size (bp)	3,279,618	2,374,946
DNA G + C content	40.76%	43.13%
Number of contigs	747	18
Contig N50	7,961	288,028
Contig L50	92	4
CDS	3,517	2,351
tRNA	59	30
rRNA	4	5
Partial CDS	0	0
Miscellaneous RNA	0	0
Chromosomes	Present	Present

Moreover, no miscellaneous RNA sequences were detected in these genomes (Table 1).

### Genomic Annotation of Strains NWUTAL1 and S52016

#### Protein Features of Strains NWUTAL1 and S52016

Annotation generated data that included hypothetical proteins and proteins with functional assignments are shown in Table 2. Proteins with functional assignment included proteins with enzyme commission (EC) numbers, those with gene ontology (GO) assignments and those mapping on KEGG pathways. Annotation with PATRIC included two types of protein families: those of the genus-specific protein families (PLfams) and those belonging to the cross-genus protein family (PGfams). The protein features of the studied strains are presented in Table 2.

**TABLE 2:** Protein features of *E. durans* NWUTAL1 and *E. gallinarum* S52016.

**Protein features**	***E. durans* NWUTAL1**	***E. gallinarum* S52016**
Hypothetical proteins	934	507
Proteins with functional assignments	2,583	1,844
Proteins with EC number assignments	833	619
Proteins with GO assignments	684	487
Proteins with pathway assignments	554	429
Proteins with PLfam assignments	3,082	2,168
Proteins with PGfam assignments	3,246	2,262

#### Subsystem Analysis of Strains NWUTAL1 and S52016 Genomes

**FIGURE 1:**
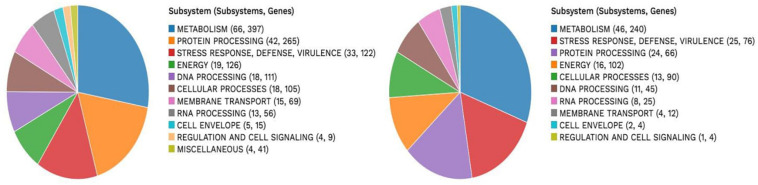
Subsystem analysis of strain NWUTAL1 **(left)** and strain S52016 **(right)**.

A subsystem refers to a set of proteins that, altogether, implement a specific biological process or structural complex (Overbeek et al., 2005). PATRIC generated an overview of the subsystems inherent to each of the studied genomes (Figure 1). Genes involved in the different cellular processes were summed up and assigned to their respective subsystems.

**FIGURE 2:**
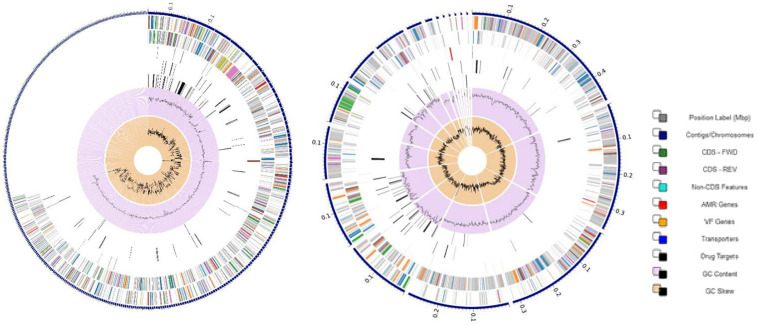
Circular graphical display of the distribution of the annotated genomes of strain NWUTAL1 **(left)** and strain S52016 **(right)**. This includes, from outer to inner rings, the contigs, CDS on the forward strand, CDS on the reverse strand, RNA genes, CDS with homology to known ARG, CDS with homology to know virulence factors, GC content and GC skew. The colors of the CDS on the forward and reverse strand indicate the subsystem that these genes belong to (see legend).

VR *E. durans* NWUTAL1 strain displayed 122 genes belonging to 33 subsystems, which play a role in stress response, defense and virulence mechanisms, compared to *E. gallinarum* strain S52016, which displayed 76 genes belonging to 25 subsystems involved in the same mechanisms. Moreover, miscellaneous genes and subsystems were not detected in strain S52016 compared to strain NWUTAL1 (Figure 1). A circular graphic display of the distribution of the genomes annotations was generated (Figure 2).

#### Genes Involved in Virulence and Antimicrobial Resistance

Analysis of the genomes revealed the presence of ARG as well as virulence genes. Both strains possessed glycopeptide resistance genes, aminoglycoside resistance genes, β-lactam resistance genes, macrolide resistance genes, tetracycline resistance genes and peptide antibiotics resistance genes, among others. ARG were identified either as genes involved in antibiotic resistance processes (such as *tet* and *bla* genes), or as genes encoding targets that may play a role in resistance mechanisms (such as *gyrA* and *gyrB* which are housekeeping genes) (Table 3).

**TABLE 3:** ARGs detected in strains NWUTAL1 and S52016.

**NWUTAL1**	**S52016**	**Resistance genes**	**Antibiotic to which resistance is conferred**	**Antibiotic group**	**Function**
	✓	*vanC1*	vancomycin	Glycopeptides	D-alanine–D-serine ligase
✓		*vanC2/C3*	vancomycin		D-alanine–D-serine ligase
✓	✓	*vanXY-C*	vancomycin		D-Ala-D-Ala dipeptidase/carboxypeptidase
✓	✓	*vanC/E/L/N*-type	vancomycin		vancomycin (or other glycopeptides) response regulator VanR
✓	✓	*macA*, *macB*	macrolides	Macrolides	macrolide-specific efflux protein *macA*, Macrolide export ATP-binding/permease protein *macB*
✓	✓	*rlmA*(II)	tylosin		23S rRNA (guanine(748)-N(1))-methyltransferase
	✓	*erm(A)*	erythromycin	macrolides, streptogramins	23SrRNA(adenine(2058)-N(6))-dimethyltransferase
✓	✓	*aac(6′)-la*	–	aminoglycosides	aminoglycoside N(6′)-acetyltransferase
✓	✓	*blaEC*	–	β-lactams	class C β-lactamase
✓		*tet(A)*	tetracycline	Tetracyclines	tetracycline resistance, MFS efflux pump
	✓	*tet(L)*	tetracycline	tetracyclines	tetracycline resistance, MFS efflux pump
✓	✓	*S10p*	tetracycline	tetracyclines	SSU ribosomal protein S10p
✓		*gyrA*	ciprofloxacine	Quinolones	DNA gyrase subunit A
✓	✓	*gyrB*	ciprofloxacine	Quinolones	DNA gyrase subunit B
	✓	*msbA*	–	Quinolones	efflux pump conferring antibiotic resistance
✓	✓	*S12p*	streptomycin	aminoglycosides	SSU ribosomal protein S12p
✓	✓	*rpoB*, *rpoC*	myxopiremine	Peptides	DNA-directed RNA polymerase β-subunit
✓		*mdfA/cmr*	multidrug efflux pump, quaternary ammonium compounds resistance		multidrug efflux pump *mdfA/cmr* (of MFS type), broad spectrum
✓	✓	*liaF, liaR, liaS*	daptomycin	peptide	membrane protein *liaF(VraT)*, specific inhibitor of *liaRS(VraRS)* signaling pathway, cell envelope stress response system *liaFSR*, response regulator *liaR(VraR)*, cell envelope stress response system *liaFSR*, sensor histidine kinase *liaS*
✓		*bcrC*	bacitracin	Polypetide	undecaprenyl-diphosphatase *BcrC* (EC 3.6.1.27), conveys bacitracin resistance
✓		*mprF*	moenomycin	phosphoglycolipid	L-O-lysylphosphatidylglycerol synthase
✓		*pgsA*	Daptomycin	peptide	CDP-diacylglycerol–glycerol-3-phosphate 3-phosphatidyltransferase
✓	✓	*ef-G*	fusidic acid	Fusidane	translation elongation factor G
✓	✓	*ef-TU*	Elfamycins		translation elongation factor Tu
✓	✓	*ddl, alr*	cycloserines		D-alanine–D-alanine ligase and Alanine racemase
✓	✓	*kasA*	isoniazid, triclosan		3-oxoacyl-[acyl-carrier-protein] synthase, KASII
✓	✓	*isotRNA*	mupirocin	carboxylic acid	isoleucyl-tRNA synthetase
✓	✓	*inhA, fabl*	isoniazid, triclosan		Enoyl-[acyl-carrier-protein] reductase [NADH]
✓	✓	*murA*	fosfomycin	fosfonic antibiotics	UDP-N-acetylglucosamine 1-carboxyvinyltransferase
✓	✓	*folA, Dfr*	trimethoprim		Dihydrofolate reductase

Some genes reported to be associated with virulence in pathogenic bacteria were noticed as follows: *pgaA* and *bopD* (biofilm formation); *cspE* (cold shock protein); *purB* (colonization factor); *ompA* (outer membrane porin); *ecbA* (cell wall surface anchor protein); and *perR* (peroxide stress regulator) for strain NWUTAL1; while *purB* (colonization factor), *ebpC* and *pgaA* (biofilm formation), *cspE* (a cold shock protein) and *ompA* as well as *ompF* (outer membrane porins) were detected in strain S52016. Moreover, a Copper/silver efflux RND transporter, outer membrane protein (*cusC*) was detected in both strains.

#### Assessment of CRISPR, Phages and Plasmids

No phages were detected in both strains. However, plasmids were detected in both strains (*E. durans* NWUTAL1 and *E. gallinarum* S52016) and aligned with reference plasmid sequences of the Enterobacteriaceae plasmids database. The plasmids detected in *E. durans* NWUTAL1 showed an identity of 99.3, 99.23, 96.54, 95.04, and 93.84% to plasmids *Incl1* (accession number: AP005147), *IncFII* (accession number: AY458016), *rep1* (accession number: NC011140), *IncFll*(pCoo) (accession number: CR942285), and *IncFIB*(AP001918) (accession number: AP001918), respectively. Comparatively, four plasmid sequences were detected in *E. gallinarum* S52016 and these demonstrated an identity of 97.32, 100, 99.38, and 95.04% to plasmids *IncFII* (accession number: AY458016), *IncFIA* (accession number: AP001918) *IncFIB* (pB171) (accession number: AB024946), and *IncFII*(pCoo) (accession number: CR942285), respectively. CRISPRFinder predicted three CRISPR on nodes 5, 307, and 729 in the genome of strain NWUTAL1. Three CRISPR were also detected on nodes 6, 1029, and 1030 in the genome of strain S52016.

Comparatively, three plasmid sequences were detected in *E. gallinarum* S52016 and these included IncFII, Incl1 and rep1. CRISPRFinder predicted three CRISPR on nodes 5, 307, and 729 in the genome of strain NWUTAL1. Three CRISPR were also detected on nodes 6, 1029, and 1030 in the genome of strain S52016.

#### Phylogenetic Assessment of Nucleotide Sequences of Strains NWUTAL1 and S52016

**FIGURE 3:**
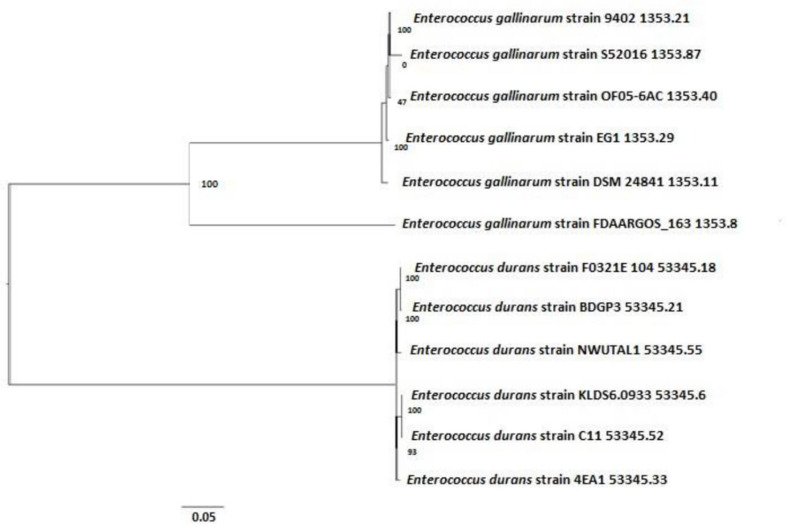
Phylogenetic tree determining the relationship between strains NWUTAL1, S52016 and other enterococci of the same species.

Based on the alignment of the 16S rDNA sequences, a high similarity was detected between strain NWUTAL1 and other strains of the same species from different sources. Similarly, strain S52016 was compared with other *E. gallinarum* strains and a high similarity was detected between them as well (Figure 3).

Moreover, sequences of the studied strains *vanC* genes were compared with that of a reference strain (accession number AF162694) that also possessed *vanC* gene. Identity scores of the *vanC* genes were 99.88 and 100 for strain NWUTAL1 and strain S52016, respectively.

## Discussion

Systematic monitoring of antibiotic usage and prevalence of antibiotic resistance among humans and animals as well as their pattern of spread in the environment, is of utmost importance as far as the management of bacterial infectious diseases and food safety are concerned (World Health Organization, 2015). The unavailability of infrastructure and resources in certain low income countries, such as insufficient financial means and underequipped hospitals with poor healthcare systems, has created gaps in the data generated worldwide, causing inefficient surveillance systems (World Health Organization, 2018). This study is in accordance with the “One Health Perspective” which was, therefore, designed to close these gaps in the antibiotic resistance surveillance data while emphasizing on the interconnections between the health and well-being of animals, humans, plants and their environment (World Health Organization, 2018).

The present investigation reveals features that are inherent to the genomes of two enterococcal isolates, namely: *E. durans* strain NWUTAL1 isolated from cattle feces of feedlots and *E. gallinarum* strain S52016 isolated from the soil of the same cattle feedlot. Data from whole genome sequence was used in the present study to assess their resistome and some virulence factors of importance. An explanation of the multidrug resistant nature of these isolates may be the ability of enterococci to adapt to their environment by incorporating, in their genomes, genetic determinants such as plasmids that harbor multiple genes, which altogether, code for resistance to either a single drug or multiple drugs (Clewell et al., 2014). Additionally, another explanation of these observations is the increased expression by enterococci of genes that code for multiple-drug efflux pumps, thus conferring to them, the ability to flush out of their cells, a wide range of antimicrobials (Miller et al., 2014). Moreover, antibiotic resistance in some cases is an inherent feature located in the chromosome, which is transmitted to progenies.

In this study, plasmids (Incl1, IncFII, rep1 and IncFIB) and most importantly, vancomycin (glycopeptide) resistance genes as well as genes of resistance to peptides, macrolides, tetracyclines, aminoglycosides, streptogramins, quinolones and β-lactams were detected in the genomes of the studied strains, with many other resistance genes to antibiotics such as bacitracin, fosfomycin, trimethoprim and fusidic acid, among others (Table 3). Vancomycin resistance can be either intrinsic or acquired. Intrinsic resistance or low-level resistance refers to the ineffectiveness of a drug due to the possession of certain genetic features, which are inherent to a species. This type of resistance is common in *Enterococcus casseliflavus*, *E. durans* and *E. gallinarum*, and *vanC* (*vanC1*, *vanC2/C3*) resistance gene confers such type of resistance (Ahmed and Baptiste, 2017). Comparatively, acquired resistance arises due to the uptake of genetic determinants either from the environment or from another bacterium. This type of resistance is common in *E. faecalis*, *E. faecium*, *E. durans* and less often, *Enterococcus avium* and *Enterococcus raffinosus*, and *vanA*, *vanB*, *vanD*, *vanE*, *vanG*, *vanL*, *vanM*, and *vanN* code for this type of resistance (Arthur et al., 1996; Ahmed and Baptiste, 2017). The vancomycin resistance genes detected in this study are involved in the intrinsic type of resistance mechanism and the same findings were reported elsewhere (Reid et al., 2001). Broadly, intrinsic glycopeptide resistance in enterococci arises when the peptidoglycan layer synthesis pathway is altered in such a way that D-alanine-D-Alanine (D-Ala-D-Ala) is replaced by D-Alanine-D-Serine (D-Ala-D-Ser). This is mediated by chromosomal attributes that render glycopeptides inactive on such strains and their offspring. Although avoparcin, a growth promoter, which was initially incriminated for the emergence of VREs, has been banned three decades ago, VREs are continuously detected worldwide as it is the case in this study. It has been proved that the emergence of VREs is due to the usage of alternative growth promoters and antimicrobials, which continue to co-select vancomycin resistance due to selective pressure (Aarestrup, 2000). As a matter of fact, the use of the macrolide tylosin in Danish pig farms was found to co-select for vancomycin resistance among enterococci (Aarestrup, 2000). Moreover, some studies have revealed that usage of erythromycin and tetracyclines in animal rearing settings accounts for the co-selection of vancomycin resistance (Aarestrup, 2000). An exhaustive list of antibiotics currently used in animal farming settings in South Africa is provided in Supplementary Table S1. This list of antimicrobials conforms to our findings as far as vancomycin resistance and the other types of ARGs detected in this study are concerned (Table 3 and Supplementary Table S1). However, there is a need to further elucidate the mechanisms through which some of these antimicrobials co-select vancomycin resistance and this is a limitation of this study.

Administration of antimicrobials to animals, either as therapeutics or as growth promoters, causes drastic changes in the gut microbiota of animals, enhancing the proliferation of drug-resistant strains such as VREs. As demonstrated by a wide range of studies, enterococci, which were initially resistant to vancomycin or any other drug may acquire more antibiotic resistance genetic determinants and additional virulence factors with plasmids upon interaction with other bacteria of the gut, giving rise to multidrug resistant isolates, which may become pathogenic and subsequently, be shed with fecal matter (Doucet-Populaire et al., 1991; Rizzotti et al., 2009; Toomey et al., 2009). This assertion may be an additional explanation of our findings. Most of the virulence factors and the ARGs detected in this study have been previously screened in other enteric isolates (Ahmed and Baptiste, 2017). The antibiotic susceptibility profiles of strains NWUTAL1 and S52016 were previously assessed against nine antibiotics (vancomycin 30 μg, tetracycline 30 μg, erythromycin 15 μg, ampicillin 10 μg, amoxicillin 10 μg, chloramphenicol 30 μg, linezolid 30 μg, ciprofloxacin 5 μg, and penicillin 10 μg) (Tatsing and Ateba, 2019). The measurement and interpretation of the zones of inhibition revealed they were both intermediate for ciprofloxacin according to the CLSI guideline (CLSI, 2017). The antibiotic resistance profile for both strains was TET^R^-AMP^R^-AMX^R^-VAN^R^-CHL^R^-PEN^R^-LIN^R^-ERY^R^. From these findings, it is suggested that presence of multidrug resistant VREs in the environment may play a significant role in the transmission and acquisition of multidrug-resistant determinants such as *vanA*, *vanB*, *vanC*, *tetK*, *tetL*, *msrA/B*, and *mefA*. With the alarming increase in antibiotic resistance globally, these new and highly sensitive techniques such as WGS may be required to mitigate the role that environment plays in the transmission of antimicrobial resistant isolates.

When soil is mixed with manure in agricultural processes, resistance genes can be transferred either vertically or horizontally to soil microbiota. Through this process, commensals and human pathogens pick up genetic determinants such as resistance genes and virulence factors with plasmids in the already polluted soil environment (Boxall et al., 2002; Ding et al., 2014; Forsberg et al., 2014; Thanner et al., 2016; Wei et al., 2019; Zhang et al., 2019). This assertion may additionally justify the detection of ARGs in the studied strains.

The effects of usage of antimicrobials in intensive rearing cannot be undermined as it has a significant impact on the environment and, consequently, on the safety of food items. Wastes from such farms may find their way into water bodies used either in irrigation processes or for recreational purposes (Economou and Gousia, 2015). Consequently, these water bodies may be contaminated with ARG that may be incorporated into the genetic make-up of their microbiota; and whenever water from such sources is used in irrigation processes, ARG and multidrug resistant isolates are propagated unto crops, which will later on be eaten by humans and animals. This will consequently lead to a never ending cycle of transmission of ARGs to commensals and other potentially pathogenic bacteria, through the food chain and various microbiomes of the environment (Acar et al., 2012; Gonzalez-Zorn and Escudero, 2012; Wei et al., 2019; Zhang et al., 2019). Moreover, even if waste from such farms were treated before being released into the environment, the problem will not be resolved since antibiotics are not completely deactivated in the process of waste treatment and after a while in the environment, they always revert to their initial active form (Ding et al., 2014). Reports of food products contaminated by multidrug resistant isolates as a consequence of extensive usage of antimicrobials in intensive animal farming are numerous (Petersen et al., 2002; Rizzotti et al., 2009; Toomey et al., 2009; Shah et al., 2012; Drissner and Zürcher, 2014; Forsberg et al., 2014; Thanner et al., 2016; Wei et al., 2019; Zhang et al., 2019). Thus, such issues that could seriously impact food safety, should be addressed promptly.

## Conclusion

The well-being of living beings depends undoubtedly on the quality of food ingested and the quality of the environment in which they thrive. Ever since antimicrobials were discovered and introduced in therapeutic regimens and intensive animal farming, the world has spawned into what many scientists call the “post-antibiotic era,” with its huge consequences on the environment and food safety. One of such consequences is the emergence of multidrug resistant strains of bacteria and the probable availability of ARG into the environment that will later on contaminate food items through previously described mechanisms. This report highlights, on a microbiological perspective, the impact of intensive animal rearing on food safety. Two multidrug resistant enterococcal strains (namely; *E. durans* strain NWUTAL1 and *E. gallinarum* strain S52016), isolated from a cattle feedlot in the North West Province, South Africa, were assessed through genomics. The detection of ARGs that code for vancomycin, tylosin, tetracycline, erythromycin, β-lactam antibiotics, quinolones, fusidic acid, bacitracin and fosfomycin, among others, in their genomes, highlights the role that intensive farming practices, such as the abusive usage of antimicrobials has on the spread and the dissemination of resistant strains such as VREs in the environment, but most importantly the risk that such strains present as far as food safety is concerned. Environment has become a pool where genetic determinants are exchanged horizontally and vertically between organisms of different ecological niches. The consequences of industrial animal rearing on food safety and subsequently on human and animal health cannot be overemphasized, thus there is an urgent need to consider alternatives to antibiotics and adopt lifestyles that are healthier and more environment-friendly.

## Data Availability Statement

The datasets presented in this study can be found in online repositories. The names of the repository/repositories and accession number(s) can be found in the article/ Supplementary Material.

## Ethics Statement

Ethical clearance was issued by the Faculty of Natural and Agricultural Sciences (FNAS) Ethics Committee. The ethics certificate number is NWU-01221-19-S9. Moreover, authorization was granted by owners of the feedlots before collection of samples.

## Author Contributions

CA: conceptualization, resources, and funding acquisition. FF, CM, CB, and CA: methodology, software, validation, and investigation. FF and CM: formal analysis. FF: writing – original draft preparation. CM, CB, and CA: writing – review and editing, supervision, and project administration. All authors contributed to the article and approved the submitted version.

## Conflict of Interest

The authors declare that the research was conducted in the absence of any commercial or financial relationships that could be construed as a potential conflict of interest.

## Supplementary Material

Supplementary Table S1 Antibiotics currently used in animal farming in South Africa.
