# Supplementary material for: Complete Genomic Analysis of VRE From a Cattle Feedlot: Focus on 2 Antibiotic Resistance
Source: Front Microbiol. 2020 Oct 15;11:571958. doi: 10.3389/fmicb.2020.571958 (PMC7593270; doi:10.3389/fmicb.2020.571958)
Supplement: Supplementary Table S1 — Antibiotics currently used in animal farming in South Africa. [file Table_1.DOCX]

**List of supplementary tables**

**Supplementary Table S1:** Antibiotics currently used in animal farming in South Africa

| Antibiotic | Treatment objective | Food animal |
| --- | --- | --- |
| Lincomycin | Feed efficiency, growth promoter and disease control | Swine, poultry |
| Tylosin | Feed efficiency and growth promoter | Poultry, cattle |
| Penicillin | Feed efficiency, growth promoter and disease control | Swine, poultry |
| Virginiamycin | Feed efficiency, growth promoter and disease control | Swine, poultry, cattle |
| Tetracyclin | Feed efficiency, growth promoter and disease control | Swine, poultry, cattle |
| Chlortetracycline | Feed efficiency, growth promoter | Swine, poultry, cattle |
| Oxytetracycline | Feed efficiency, growth promoter | Cattle |
| Erythromycin | Disease control | Swine, poultry, cattle, sheep |
| Bacitracin | Feed efficiency, growth promoter | Swine, poultry, cattle |
| Lasalocid | Feed efficiency, growth promoter | Cattle |
| Monensin | Feed efficiency, growth promoter | Cattle |
| Fluoroquinolones | Disease control | Cattle, poultry |
